# Paucisymptomatic opisthorchiasis in a non-endemic country: a neglected differential diagnosis?

**DOI:** 10.1007/s15010-026-02853-4

**Published:** 2026-06-01

**Authors:** S. Dirik, C. Boesecke, S. Breitschwerdt, S. M. Fünger, A. Hoerauf, M. P. Hübner, G. J. Rieke, J. K. Rockstroh, J. C. Wasmuth, H. Wobser, D. Benke

**Affiliations:** 1 Faculty of Medicine, Department of Infectious Diseases and Clinical Microbiology, Ege University, Izmir, Turkey; 2 Department of Medicine I, University of Bonn, University Hospital Bonn, Bonn, Germany; 3 Institute for Medical Microbiology, Immunology and Parasitology, University of Bonn, University Hospital Bonn, Bonn, Germany; 4 German Center for Infection Research (DZIF), Partner Site Bonn-Köln, Bonn, Germany

**Keywords:** Opisthorchiasis, *Opisthorchis felineus*, Stool microscopy, Serologic test, Gallbladder ultrasonography neglected tropical disease

## Abstract

**Case presentation:**

We report a case of opisthorchiasis in a 71-year-old woman with intermittent upper abdominal pain, whose cause had remained unclear for a long time. She grew up in Kazakhstan and moved to Germany more than 30 years ago. After positive serological testing for *Opisthorchis* during a recent visit to her home country, she presented at our clinic for further evaluation. Stool examination confirmed the presence of *Opisthorchis* eggs. The symptoms quickly resolved after treatment with praziquantel. Abdominal sonography revealed adenomyomatosis of the gallbladder fundus and multiple gallbladder polyps, most likely a consequence of chronic inflammation caused by the parasite. Sonographic surveillance was recommended with regard to a potentially increased risk of cholangiocarcinoma.

**Discussion and conclusions:**

No certified serological test for opisthorchiasis is available in Germany and molecular or antigen-based tests for this infection are also largely unavailable. Diagnosis therefore has to rely on stool microscopy which requires the examination of multiple samples to ensure adequate sensitivity. Diagnosis is further complicated as symptoms are often nonspecific and liver flukes are usually not among the initial differential diagnoses. Missing the diagnosis, however, carries the risk of serious complications. Global travel and migration as well as recent autochthonous cases in the European Union underscore the need for increased vigilance for opisthorchiasis even in non-endemic areas.

## Introduction

*Opisthorchis* spp. are trematodes transmitted through the consumption of raw or undercooked freshwater fish [1]. They are endemic in parts of Asia and Eastern Europe. *Opisthorchis felineus* mainly occurs in Kazakhstan (esp. in the north and north-east), Russia (esp. in Western Siberia) and Ukraine (esp. in the north-east/ Sumy oblast) [1]. *Opisthorchis viverrini* is predominantly found in Southeast Asia, particularly in Thailand, Laos, Vietnam and Cambodia [1].

Together with the closely related *Clonorchis sinensis*, which is mainly found in China, Korea, Vietnam and Russia, they affect approximately 27 to 46 million people worldwide [1, 2]. Adult worms reside in the biliary ducts of humans or other mammalian hosts, including cats and dogs. Eggs are excreted in the stool and develop into cercariae when taken up by a snail, the first intermediate host. Freshwater fish represent the second intermediate host harbouring metacercariae, the infective stage for humans [1].

Infections are often mild or asymptomatic [3]. Acute infection can lead to nonspecific symptoms like abdominal pain, nausea, diarrhoea and fever [1]. Chronic infection can cause ongoing abdominal discomfort and increase the risk of cholangitis, cholecystitis, pancreatitis or liver cirrhosis [1, 4]. Moreover, *O. viverrini* and *C. sinensis* are classified as group 1 carcinogens by the *International Agency for Research on Cancer* due to their strong association with cholangiocarcinoma [5]. For *O. felineus*, carcinogenicity has been less studied and evidence is heterogeneous [4, 6–8].

Diagnosis is usually made by microscopic identification of helminth eggs in stool samples. Stool microscopy, however, has limitations as sensitivity might be reduced in cases with low parasite load or due to intermittent egg excretion. It will also be false-negative during the prepatent period before eggs are produced and is highly dependent on the experience of the examiner [9]. Other methods, if available, are therefore a helpful complement to stool microscopy. These include serological tests to detect specific antibodies in serum and molecular tests to detect parasite DNA in stool. Laboratory abnormalities like eosinophilia and elevated liver or cholestatic parameters can support diagnosis, but may be absent [9]. Imaging can also be suggestive of opisthorchiasis. In heavy infections, worms in the gallbladder can occasionally be detected by ultrasonography [10]. Sometimes, they can be visualised by endoscopic retrograde cholangiography as small filling defects within the bile ducts [10].

## Case presentation

A 71-year-old woman with intermittent upper abdominal pain presented at our clinicin Bonn, Germany, after serological testing during a recent stay in Kazakhstan had detected the presence of *Opisthorchis*-specific antibodies, to further evaluate the possibility of opisthorchiasis as the underlying cause of her complaints. The symptoms had been present for years with fluctuating intensity. They had worsened on the third day of her stay, prompting her to seek medical attention. As far as can be ascertained from the documentation provided, *Opisthorchis* serology was requested based on the clinical presentation and abnormal findings on abdominal ultrasound. It yielded positive IgG and negative IgM antibody results. A serological test for *Giardia lamblia* was negative. Treatment had not yet been initiated. She had travelled to Aksay in north-western Kazakhstan. An extended travel history revealed trips to Chișinău, Moldova, and a trip to Venice, Italy. In Germany, the patient’s primary country of residence, the cause of her complaints had previously remained unclear. The patient reported that gastroscopy and colonoscopy were unremarkable and that an attempt to relieve symptoms with proton pump inhibitors had been unsuccessful. She had a history of hypothyroidism and depression, for which she was taking levothyroxine, sertraline and quetiapine. Her bowel movements were normal and she had no fever or weight loss. The patient reported that she frequently ate fish and enjoyed fishing as a hobby. She did not usually eat raw freshwater fish, but could not rule out consumption of undercooked carp. Of note, she had not consumed fish during her last stay in Kazakhstan prior to serological testing.

**Fig. 1:**
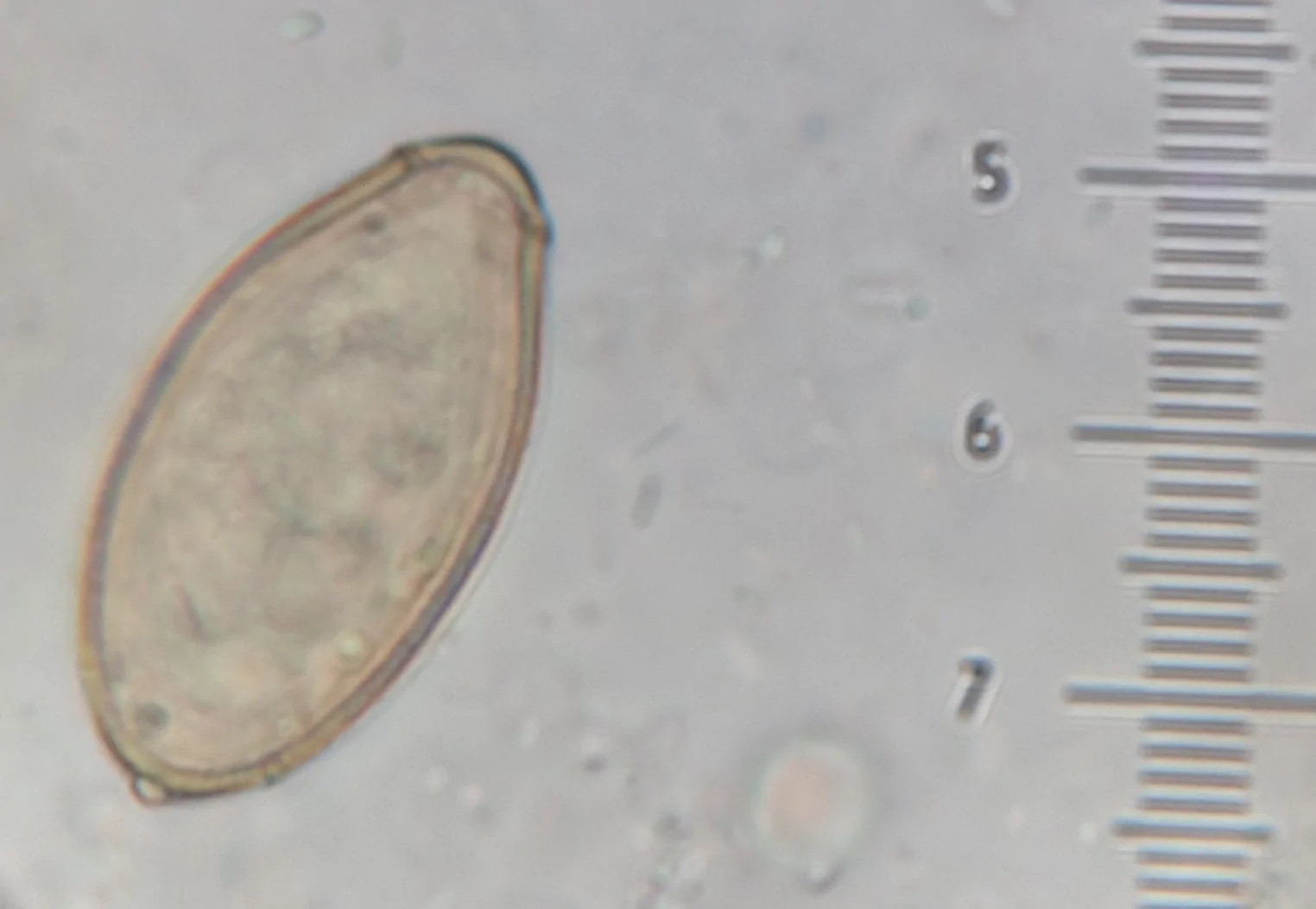
Stool microscopy showing an egg of *Opisthorchis felineus* (length approx. 30 μm, original magnification x40), with the operculum at the top and an abopercular knob at the opposite end. Several eggs were identified, indicating a modest infection intensity

**Fig. 2:**
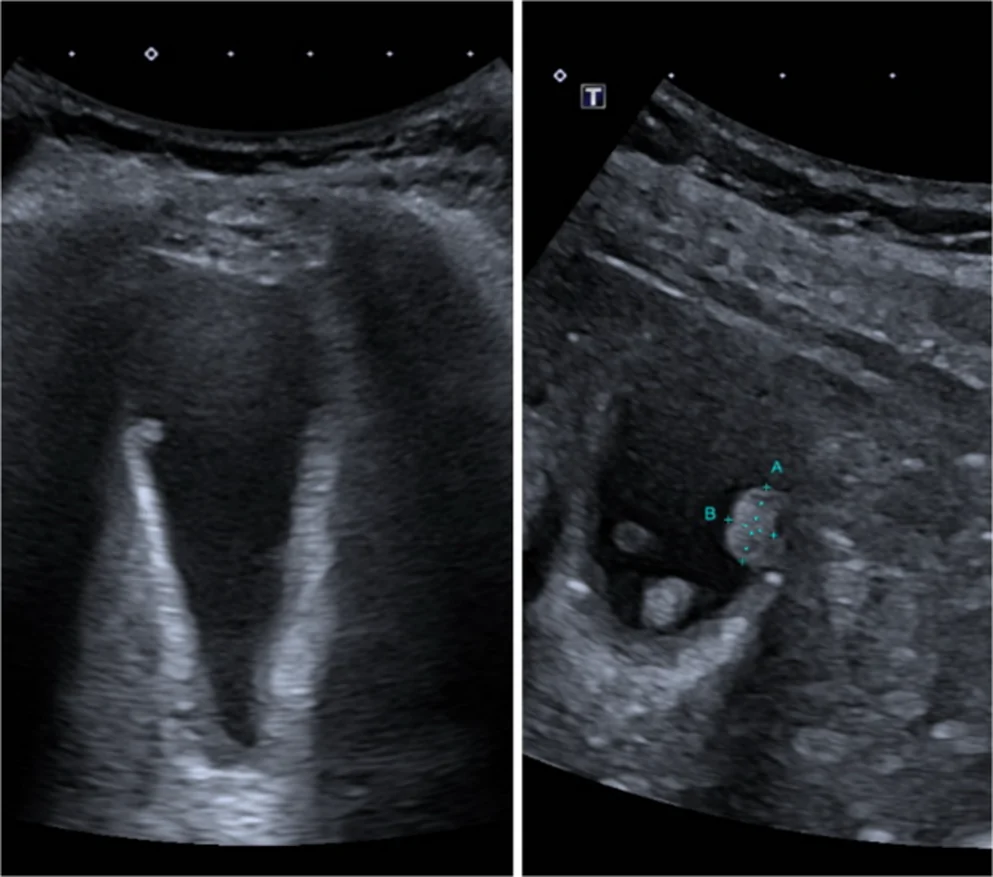
Gallbladder ultrasonography. Adenomyomatosis of the gallbladder fundus (left image) and multiple gallbladder polyps (approx. 7 identified), the largest measuring 7 × 4 mm (right image)

Except for a slightly elevated creatine kinase of 222 U/l (reference range 0–170 U/l), laboratory findings from our clinic were unremarkable and in particular did not show increased transaminases, cholestatic parameters, C-reactive protein or eosinophils. Parasitological stool examination identified the presence of helminth eggs compatible with *Opisthorchis/ Clonorchis* (Fig. 1). Due to the geographic distribution of liver flukes and the patient’s history, we assumed infection with *Opisthorchis felineus.* Treatment with praziquantel (25 mg/kg three times daily for 3 days) led to complete and lasting resolution of symptoms. Abdominal ultrasonography showed adenomyomatosis of the gallbladder fundus and multiple polyps of the gallbladder, the largest of which measured 7 × 4 mm (Fig. 2). It also revealed pancreatic lipomatosis and a small cyst in the pancreatic body, with no evidence of ductal connection. Follow-up sonography after 6 months showed stable findings, and further sonographic surveillance was recommended.

## Discussion

This case illustrates how the diagnosis of opisthorchiasis with mild to moderate symptoms can easily be overlooked for a long time in a non-endemic setting. Without serology performed in Kazakhstan, where opisthorchiasis is a more common differential diagnosis, it might have remained undiagnosed. Our assumption that the patient had chronic opisthorchiasis rather than an acute (re)infection is based on the fact that she reported not having eaten fish during her most recent stay in Kazakhstan and that her long-standing symptoms resolved completely after treatment with praziquantel. As *Opisthorchis* can survive in the human host for more than 10 years [9], it seems likely that the infection occurred during one of her earlier visits to Kazakhstan or Moldova, though acquisition in Germany, where the patient also went fishing, cannot be ruled out.

In a non-endemic setting like Germany, opisthorchiasis is an unusual cause of upper abdominal pain. Limited clinician awareness therefore is the most likely reason for missed *Opisthorchis/ Clonorchis* diagnoses. The restricted availability of serological, molecular or antigen-based tests represents another diagnostic hurdle, as stool microscopy is labour-intensive and impractical as a screening tool, especially when mild and nonspecific symptoms imply a low pre-test probability. Although not performed in the case presented here, serology for *Fasciola* spp. is more commonly available and might be helpful in the context of suspected *Opisthorchis* infection, due to potential cross-reactivity [11].

If assessment of upper abdominal pain remains unremarkable for common aetiologies, our case suggests that it can be worth requesting stool microscopy in patients with a history of having lived in a country endemic for liver flukes who eat fish. We recommend at least five, better ten, stool samples from different days for microscopic examination, to achieve sufficient sensitivity. In view of current migration trends, it is worth mentioning that *Opisthorchis felineus* is also endemic in parts of Ukraine [1, 12]. Although opisthorchiasis in Europe is primarily associated with travel or migration, local transmissions of *Opisthorchis* have recently been described in Italy, where serology and molecular testing helped identify cases of asymptomatic patients with negative stool examination, in the context of outbreak investigations [13]. In Germany, no proven autochthonous cases of opisthorchiasis in humans have been described so far; the parasite has, however, been reported in animals [14, 15]. Climate change may influence the future epidemiology of opisthorchiasis by altering transmission dynamics of the parasite or abundance of intermediate hosts; robust models need to be developed to better predict these changes [16].

Even in this case with relatively mild symptoms, we could observe sonographic abnormalities of the gallbladder that we consider to be a likely result of chronic inflammation caused by the trematode. Although association with cholangiocarcinoma is less clear for *Opisthorchis felineus*, the findings underline the potential for complications arising from chronic infection with liver flukes. Here, follow-up ultrasound of the gallbladder polyps was indicated in any case, due to their risk of malignant transformation, irrespective of the underlying aetiology.

Apart from the already mentioned unavailability of diagnostic tests and low awareness for the disease, management of opisthorchiasis is further complicated by the recent end of praziquantel supply in Germany. The need to import the medication or to organise extemporaneous compounding by specialised pharmacies can delay therapy and alternatives like albendazole have been described as less effective [13, 17]. Our case therefore also demonstrates that opisthorchiasis, classified by the World Health Organization as a neglected tropical disease, remains neglected even in high-resource healthcare settings.

## Data Availability

No datasets were generated or analysed during the current study.
